# Chemical and Physical Properties of African Catfish (*Clarias gariepinus*) Fillet Following Prolonged Feeding with Insect Meal-Based Diets

**DOI:** 10.1155/2023/6080387

**Published:** 2023-08-29

**Authors:** Askale Gebremichael, András Szabó, Zsuzsanna J. Sándor, Zoltán Nagy, Omeralfaroug Ali, Balázs Kucska

**Affiliations:** ^1^Department of Freshwater Fish Ecology, Institute of Aquaculture and Environmental Safety, Hungarian University of Agriculture and Life Sciences, Guba S. u. 40., 7400, Kaposvár, Hungary; ^2^Department of Animal Science, Mizan-Tepi University, 260 Mizan Aman District, Mizan Teferi, Ethiopia; ^3^Agribiotechnology and Precision Breeding for Food Security National Laboratory, Institute of Physiology and Nutrition, Department of Physiology and Animal Health, Hungarian University of Agriculture and Life Sciences, Guba S. u. 40., 7400, Kaposvár, Hungary; ^4^Research Centre of Aquaculture and Fisheries, Institute of Aquaculture and Environmental Safety, Hungarian University of Agriculture and Life Sciences, Anna-Liget. u. 35., Szarvas 5540, Hungary

## Abstract

A 25-week experiment was undertaken to explore the effect of partial replacement of dietary fishmeal (FM) with black soldier fly meal (*Hermetia illucens*) (BS), mealworm meal (*Tenebrio molitor*) (MW), and a 1 : 1 mixture of both insect meals (BSMW) on fillet quality in African catfish (*Clarias gariepinus*). A total of 96 fish with an average initial body weight of 248 ± 28 g were stocked into a recirculating aquaculture system and fed in four different dietary groups (control, BS, MW, and BSMW). No mortality was recorded in any of the groups. At the end of the feeding period, 24 fish (*n* = 6 for each treatment, weight between 690 and 822 g) were used for analysis. There was no alteration in filleting yield or other slaughter indices within experimental groups, except the hepatosomatic index. Among quality attributes, pH 24 hr postmortem exhibited a significant difference (*p* < 0.05). In respect of the fatty acid profile, the *n*-6/*n*-3 ratio ranged between 1.17 and 1.40 but was not significantly modified by the partial replacement of FM. Similarly, the proximate composition of the fillets was not significantly different between the control and experimental diet groups. The ratio of polyunsaturated fatty acid to saturated fatty acids ranged between 0.67 and 0.79 in the fillets, without significant differences between groups. The atherogenic index was increased in the BS group, as compared to the others; however, the thrombogenicity index of fillets was not significantly affected. Similarly, the conventional quality traits of the fillet, such as cooking, drip, and thawing losses, did not differ within treatments. This study demonstrates that the dietary inclusion of black soldier fly and/or mealworm meals used for African catfish at the tested inclusion level has negligible impact on fillet properties.

## 1. Introduction

Nowadays, the aquaculture sector is growing rapidly in an effort to satisfy the high protein demand of the projected 9 billion human population around the globe by 2050. Fish require a higher proportion of protein sources in their diet than any other farmed animal and efficiently convert it to skeletal muscle and used as a meat source. Fishmeal is an excellent source of dietary protein in the fish diet, but its supply is limited; it is unsustainable and unaffordable due to overfishing pressure and high demand [1–3]. Insects are, in contrast, eco-friendly, sustainable in supply, and provide a biologically valuable protein source in fish nutrition due to their protein, fat, and mineral contents: crude protein content ranges from 9.3% to 76% [4–6], fat ranges from 7.9% to 40%, and macro and micronutrients are fair [7–9]. Therefore, insects can be a promising novel alternative protein source to replace unsustainable and unaffordable fishmeal in the fish diet.

The African catfish (*Clarias gariepinus*) is one of the major fish species cultured in Africa. It has also been introduced into aquaculture in different parts of the world, including the Netherlands, Hungary, much of South-East Asia, and East Asia [10]. The species is also one of the most important individual commercial freshwater fish in many parts of Africa [11, 12]. This species can be cultivated in areas with a tropical climate, areas with access to geothermal waters, or with the use of heated recirculating water systems. It is considered a very hardy fish in aquaculture terms and can be densely stocked in low-oxygen waters, making it ideal for culture in areas with a limited water supply.

Since dietary composition is a major determinant of the nutritional quality of fish flesh [13, 14], numerous reports are available on African catfish slaughter traits and fillet quality [15, 16]. However, there are limited reports related to the effect of dietary insect meal on the quality of fish flesh. This study aimed to evaluate the effects of 10% dietary inclusion of black soldier fly and mealworm meal as partially replacing fishmeal on the slaughtering yield and on the meat quality parameters of African catfish.

## 2. Materials and Methods

### 2.1. Experimental Diets

One control and three experimental diets were formulated (Table 1) using locally produced ingredients and two types of insects available on the market. The control diet (C) contained 20% fishmeal, a common ingredient in catfish feeds. In each experimental diet, half of the fishmeal (10%) was either replaced with black soldier fly meal (BS), yellow mealworm meal (MW), or a 1 : 1 mixture of both insect meals (BSMW). The experimental diets were set to be iso-nitrogenous and iso-energetic (Table 2). MW was purchased from Berg and Schmidt Pt Ltd. Singapore and imported by Hecron-Agro Kft. Hungary, while BS meal was supported by Agroloop Ltd. Netherlands. Both were defatted and ready for use in the feed extrusion process. The feed ingredients (Table 1) were thoroughly mixed to form a homogenous blend, moistened with water (200 mL kg^−1^) then extruded with a single screw extruder (Abrazive, Hungary) to produce 6 mm sinking pellets, which were dried in an oven at 55°C (Pol-Eko, Wodzislaw Slaski, Poland). The chemical compositions of insects used in feeds are similar to data presented by Sándor et al. [17].

Chemical composition analysis showed that experimental diets formulated were isoenergetic (19.05–19.94 kJ g^−1^) and isonitrogenous (45.11%–45.97%) (Table 2). Crude fiber and acid detergent fiber (ADF) levels of the diets were raised by the inclusion of insect meal. Furthermore, the ADF was significantly higher in the treatments with MW inclusion (MW and BSMW). The crude ash level of the control diet reached 10.6%, while 8.9%–9.4% was found in the experimental feeds. The fatty acid profiles of the feeds differed in saturated fatty acids, especially in lauric acid (C12 : 0), when a high level in BS-containing feed was detected (Table 3). The levels of eicosapentaenoic acid (EPA, C20 : 5*n*-3), docosapentaenoic (DPA, C22 : 5*n*-3), and docosahexaenoic acid (DHA, C22 : 6*n*-3) decreased with increasing dietary fishmeal (FM) replacement, contributing to a higher *n*-6/*n*-3 ratio of the feed. This ratio was highest in BS-containing feeds compared to the MW diet.

### 2.2. Experimental Setup and the Fish

**Figure 1:**
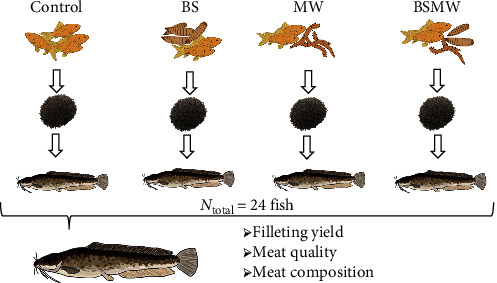
Schematic diagram of the experimental setup and the measured parameters at the slaughter.

A 25-week experiment was carried out at the Hungarian University of Agriculture and Life Sciences, Kaposvár Campus, in a recirculating aquaculture system. A total of 96 fish with an average initial body weight of 248.7 ± 0.08 g were randomly assigned to four dietary groups (control, BS, MW, and BSMW), in each group 24 fish per tank (Figure 1).

The fish were fed 1.75% of their biomass weight twice a day by hand distribution at 8:00 am and 4:00 pm. The biomass weight was measured on a two-weekly basis in order to adjust the daily feed proportion. The water quality was regularly analyzed for temperature, NO_3_, NO_2_, dissolved oxygen, and pH, and the minimum and maximum values were 25.4–25.6°C, 20–44.8, 0.14–0.24, 3.9–5.0, and 7.5–7.8 mg L^−1^, respectively.

### 2.3. Sample Collection and Physical Parameter Analysis

At the end of the experiment, six fish from each group, totally 24 fish were sacrificed by manual stunning method following administration of anesthesia (2-phenoxyethanol, Merck Sigma Aldrich, Schnelldorf, Germany). The fish were dissected, internal organs removed, and slaughter traits measured. The slaughtering indices were determined as the proportion of a certain part of carcass weight to the whole-body mass, as explained in Section 2.6. For fatty acid and proximate quality analysis, the fillet samples were stored at −20°C until analysis. Muscle acidification and pH were measured at 45 min postmortem (pH 45ʹ) and after 24 hr. (pH 24 hr) with the use of a portable Testo 205 precision pH meter (Testo AG, Lenzkirch, Germany). To determine the cooking loss, 100 g of fillet samples were closed into sealed bags and cooked at 75°C for 20 min. The exudate weight, as expressed as the percentage of the initial sample weight, was referred to as cooking loss. The thawing loss was determined in the same manner, i.e., 50 g samples were frozen at −20°C and thawed to room temperature after 2 days. The dripping loss was determined using a 100 g sample cut from the carcass and immediately weighed. The samples were placed in the netting and then suspended in an inflated bag, ensuring that the sample did not make contact with the bag. After 24 hr storage period at 4°C, samples were again weighed [18].

### 2.4. Feed and Fillet Composition Analysis

The proximate composition of diets and fillets of 24 fish individuals was analyzed by standard methods of the AOAC [19]. The moisture content was determined by drying the samples in an oven at 105°C until a constant weight was achieved and then cooling in a desiccator. For crude ash determination, samples were weighed and placed in a muffle furnace at 550°C for 8 hr. The Soxhlet ether method was used for crude fat analysis. The Kjeldahl method was used to determine the nitrogen content of the samples, and based on its content, the crude protein content was calculated. The gross energy was determined by a Parr Instruments 6400 calorimeter bomb (Moline, Illinois, USA) calibrated with benzoic acid. The crude fiber and acid-soluble fiber were determined with a Fibretherm analyzer (Gerhardt, Königsberg, Germany).

For fatty acid measurements, the fish fillets and diets were individually homogenized (IKA T25 Digital Ultra Turrax, Staufen, Germany) in a 20-fold volume of chloroform: methanol (2 : 1 v/v), and complex lipid content was extracted [20]. Solvents were ultrapure-grade (Sigma-Aldrich, St. Louis, MO, USA), and 0.01% w/v butylated hydroxytoluene was added to prevent fatty acid oxidation. This latter fraction was evaporated to dryness under a nitrogen stream and was trans-methylated with a base-catalyzed NaOCH_3_ method [21]. Fatty acid methyl esters were extracted into 300 *μ*L ultrapure *n*-hexane for gas chromatography (AOC 20i automatic injector; Shimadzu 2030, Kyoto, Japan) equipped with a Phenomenex Zebron ZB-WAXplus capillary GC column (30 m × 0.25 mm ID, 0.25 *μ*m film, Phenomenex Inc., Torrance, CA, USA) and a flame ionization detector. Characteristic operating conditions were: injector temperature: 220°C; detector temperature: 250°C; helium flow: 28 cm s^−1^. The oven temperature was graded: from 60 (2 min hold) to 150°C, from 150 to 180°C: 2°C min^−1^ and 10 min at 180°C, from 180 to 220°C: 2°C min^−1^ and 16 min at 220°C. The make-up gas was nitrogen. The calculation was performed with the LabSolutions 5.93 software, using the Post Run module (Shimadzu, Kyoto, Japan), with manual peak integration. The identification of fatty acids was performed based on the retention time of a CRM external standard (Supelco 37 Component FAME Mix, Merck-Sigma Aldrich, CRM47885). Fatty acid results were expressed as the weight percentage of total fatty acid methyl esters.

### 2.5. Ethical Issues

Maximum efforts were made during measurements to minimize the fish's suffering.

### 2.6. Calculations and Statistical Analysis

In order to determine the biometric indexes and culinary traits, the following parameters were measured and calculated at the end of the trial:

  Hepatosomatic index (HSI%) = 100 × liver weight (g)/body weight (g) [22],
  Viscera somatic index (VSI%) = 100 × visceral weight (g)/body weight (g) [22],
  Filleting yield (%) = 100 × fillet without skin (g)/body weight (g) [18],
  Eviscerated slaughter value (%) = 100 × eviscerated body weight (g)/body weight (g) [22],
  Trunk with skin (%) = 100 × trunk with skin (g)/body weight (g),
  Skinned trunk (%) = 100 × trunk without skin (g)/l body weight (g),
  Dripping loss (DL) (%) = 100 × ((raw fillet weight (g) − raw fillet weight after 24 hr (g))/raw fillet weight (g)) [18],
  Thawing loss (TL) (%) = 100 × ((raw fillet weight (g) − thawed fillet weight (g))/raw fillet weight (g)) × 100 [18],
  Cooking loss (CL) (%) = 100 × ((raw fillet weight (g) − cooked fillet weight (g))/raw fillet weight (g)) [18].

Calculations of the atherogenic index and thrombogenic index were based on formulas used in our published work by Ali et al. [23] as follows:

  Atherogenic index = (C12 : 0 + (4 × C14 : 0) + C16 : 0)/(total *n*-6 FA + total *n*-3 FA + total MUFA);
  Thrombogenic index = (C14 : 0 + C16 : 0 + C18 : 0)/((0.5 × total MUFA) + (0.5 × total *n*-6 FA) + (3 × total *n*-3 FA) + total *n*-3 FA/total *n*-6 FA).

For statistical analysis, all the data were checked for normality with Shapiro–Wilk normality test and homogeneity of variances using Levene's tests. Then, data were analyzed with one-way analysis of variance using SPSS version 20 at a confidence interval of 95%. The significant difference was considered for a *p*-value < 0.05. Means were compared using post hoc Tukey's multiple comparison test.

## 3. Results

### 3.1. Slaughtering Indices and Physical Characteristics of the Fillets

After 25 weeks of feeding, the survival was 100% in all dietary groups. Final body weights of fish involved in the determination of slaughtering yield (eviscerated %, trunk with skin %, trunk without skin %, and filleting yield %), proximate composition, and quality traits ranged between 690 and 822 g. It was found that slaughtering indices did not differ (*p* > 0.05) between control and treatments (Table 4). However, the hepatosomatic index was significantly increased in the BS diet group. Cooking, thawing, and dripping loss values did not differ (*p* > 0.05) among the dietary groups and ranged between 17.19%–23.61%, 5.06%–6.06%, and 2.78%–3.90%, respectively (Table 4). The acidification value (pH) of the fillet after 45 min postmortem did not exhibit a significant difference (*p* > 0.05) in any of the different diet groups. However, the pH 24 hr postmortem showed significantly the highest pH of 6.61 in the BS diet group (*p* < 0.05) (Table 4).

### 3.2. Fillet Proximate Composition and Fatty Acid Profile

After the feeding period, the proximate composition of catfish fillets is presented in Table 5. The crude protein varied between 18.05% and 18.82% wet weight, while the fat content was between 3.45% and 3.90%. However, none of the determined parameters provided a significant difference among treatments.

The lauric acid (C12 : 0) proportion was significantly increased in the BS and MW groups, while the heptadecanoic acid (C17 : 0) proportion was highest in control and significantly lowest in the MW groups (Table 6) fillet samples. Among polyunsaturated fatty acids (PUFA), stearidonic acid (C18 : 4*n*3) proportion was significantly increased in the MW group compared to the BS group. A slight increase in the total saturated (total SAT) and monounsaturated (total MUFA) FA proportion was observed as fishmeal was partially replaced by black soldier fly or mealworm meals. The *n*-6/*n*-3 ratio ranged between 1.17 and 1.40 but was not significantly modified by partial replacement of FM, similar to the proximate composition of the fillets. The ratio of total PUFA to total SAT ranged between 0.67 and 0.79 in the fillets, without significant differences between groups. The atherogenic index was significantly highest in fillets from the BS diet group.

## 4. Discussion

There are similar findings where BS and MW-containing diets were applied at different inclusion levels with species like Jian carp (*Cyprinus Carpio* var. Jian) [24], rainbow trout (*Oncorhynchus mykiss*) [25], common carp (*Cyprinus carpio*) [26, 27], African catfish [28, 29], and Siberian sturgeon (*Acipenser baerii*) [30]. However, in most cases, juvenile fish is the targeted age category for such nutritional trials. In respect of the evaluation of the market-size fish quality, Iaconisi et al. [31] confirmed the absence of significant differences in slaughter traits (filleting yield) in blackspot seabream fed diets supplemented with full-fat mealworm larvae meal replacing up to 50% of FM compared with the control group. A review study [32] reported that the results of the morphometric and the slaughter traits presented so far are quite heterogeneous; several found significant impacts, while in other cases, effects on the morphometry and dressing yield could not be detected. The slaughtering indices of our table-sized fish were not different between the control and treatments (Table 4); however, in BS and BSMW treatments seem to be higher compared to MW or the control group.

The cooking loss values determined in the present study for BSMW and BS treatment were also higher (21.90% and 23.61%) compared to earlier findings [16] when 19.8%–19.9% values were obtained for African catfish with a 2 kg average body weight or from the results obtained by Szabó et al. [15] (7.7%–11.1%) when the African catfish adults were fed with different vegetable oil inclusion diets. Similarly, the dripping loss was higher in our case, while the thawing loss was similar to published data [16] (1.7%–2.2% and 5.9%–8.1%, respectively). These alterations are almost related to the different body weights of the fish involved in these investigations. Similarly, the absence of significant differences in cooking, thawing, and dripping loss parameters was reported in rainbow trout fed up to a 100% (150 g kg^−1^) replacement rate of fishmeal with BS meal [33]. Furthermore, Iaconisi et al. [34] found no marked difference in cooking loss of rainbow trout-fed diets supplemented with up to 50% full-fat MW larvae meal compared to fish fed on a control diet.

Unlike mammalian and poultry meat, with a rapid postmortem drop in pH, reaching a peak level at about 5.5–5.8, fish meat is poor in glycogen, and the postmortem pH decrease is significantly gradual, but most of the fish species do not exhibit ultimate pH values lower than 6.0–6.2 [35–37]. In the present study, pH 45ʹ was significantly higher than pH 24 hr in most groups, except BS (Table 4). The acidification (pH) of the fish fillet is an indicator of stress exposure in fish prior to slaughter. The pH has a significant impact on the tenderness state of meat [38]. The antemortem handling of fish and the stress suffered before and during slaughtering have a great impact on the quality of the final product, including low pH [39, 40]. These studies reported that the fillet pH was significantly lower in the stressed fish than in the rested group until 18 hr postharvest. In the present study, the fish fed BS meal at 100 g kg^−1^ inclusion level showed the highest value at pH 24 hr postmortem, and this might relate to the antemortem stress tolerance of the fish. The black soldier fly meal is rich in lauric acid (12 : 0), which is the principal constituent of the medium-chain triacylglycerols (MCTs) and has a role in stress resistance and immune system building, possibly indicative of the highest pH recorded [40, 41]. MCTs, due to the rapid absorption and oxidation, are able to inhibit lipid deposition in the body [42], which makes their utilization in fish nutrition advantageous as well.

There is supporting evidence based on previous studies about the possible or no adverse effect of insect meal on the proximate quality of fish fillet, e.g., defatted MW meal on European perch [43], BS on Nile Tilapia [44], BS on Atlantic salmon [45] and BS on Jian carp [24]. Generally, the fillet of African catfish has a high total crude protein content (16.91%–17.90%) and a comparatively low-fat content (3.95%–7.57%) [46]. Considering lipid content, fish meat can be classified into lean (<2% fat), low-fat (2%–4%), medium fat (4%–8%), and fat (>8%) categories. In the present study, the fillet protein content was above 18% in all groups, and the fat content was set between 3.45% and 3.90%; thus, the fillet of African catfish with body weight lower than 1 kg is considered as high-protein and low-fat meat.

The partial replacement of fishmeal with BS and MW caused only a slight alteration in the total PUFA proportions. According to the reports of Belforti et al. [47], the proportions of EPA, DPA, and DHA in rainbow trout fillets decreased with increasing levels of mealworm meal inclusion. Similarly, it was reported that the whole body and fillet EPA, DPA, DHA content, and *n*-6/*n*-3 fatty acid ratio decreased as the inclusion level of mealworm meal increased in the diets of juvenile European Sea bass and Nile tilapia [48, 49]. In addition, Zhou et al. [24] confirmed the significant reduction of EPA and DHA in the Jian carp body when the fish were fed up to 140 g kg^−1^ with black soldier fly larvae. It is quite advantageous that the inclusion of originally PUFA-poor insect meals did not significantly compromise the biological value of the diets and did thus not worsen the PUFA levels of the catfish meat in our case. However, fatty acid profile modification of insects is possible with the manipulation of the rearing substrate [48]. On the other hand, insects are generally rich in saturated fatty acids [50, 51]. In particular, BS larval fat consists mainly of C12 : 0, C14 : 0, and other saturated fatty acids [52, 53]. However, in the current study, the higher lauric acid content in BS resulted in a decreasing tendency for total PUFA as a consequence of significant proportional reductions in C18 : 4*n*3 and C22 : 1*n*11. At the same time, it did not significantly affect the fillet crude fat level (Table 5) as compared to the fish fed on the control diet. So, overall, it would not be a quality issue [45]. Jian carp and rainbow trout high in lauric acid did not reduced the lipid level of fish whole body [54, 55]. On the other hand, as we mentioned in proximate quality parameters, the presence of high lauric acid positively impacted the postmortem pH. The other factor might be the effect of insect meal-originated chitin on fish feed intake. Meanwhile, the indigestibility of chitin affects nutrient utilization by absorbing lipids and bile in the gastrointestinal tract, and thus, possibly decreasing or at least limiting lipid digestion and absorption [56].

The *n*-6/*n*-3 ratio of fish fillets in the present study ranged between 1.17 and 1.40, without displaying any alteration among groups. According to health recommendations for humans, the *n*-6/*n*-3 ratio should be lower than 4, thereby reducing the incidence of chronic food-related illnesses [57]. In our study, the atherogenic index was significantly high at partial replacement of fishmeal with black soldier flies meal. This finding is a result of the high lauric acid proportion of black soldier fly meal. However, our finding is within the range (0.33%–0.70%) determined in several fish species by Łuczyńska et al. [58]. The thrombogenic index did not differ significantly with fish meal replacement with any of the insects investigated. The recommended polyunsaturated to saturated fatty acids ratio is to be higher than 0.4 in animal products in order to reduce the risk of cardiovascular, autoimmune, and other chronic diseases [59]. Interestingly, in the present investigation, this ratio ranged between 0.66 and 0.79 and was not significantly affected by the partial replacement of fishmeal.

## 5. Conclusions

This study demonstrated that 50% partial replacement of fishmeal with black soldier fly meal and/or yellow mealworm meal in the catfish diets did not affect fillet physical and chemical properties, such as culinary technology traits, proximate composition, and fatty acid profile, in a 25-week setting. However, fillet organoleptic profile or mineral nutrient content, which are considered important factors from the consumer perception viewpoint, were not assessed in the frame of this study. In conclusion, partial inclusion of black soldier fly and mealworm meal seems to be suitable for meat-producing African catfish without compromising product quality.

## Figures and Tables

**Table 1:** Composition of control and experimental feeds (%).

Ingredients (%)	Control	BS	MW	BSMW
^1^Fishmeal (FM)	20	10	10	10
^2^Mealworm meal (MW)	0	0	10	5
^3^Black soldier fly (BS)	0	10	0	5
^4^Soy protein concentrate	15.0	15.0	15.0	15.0
Wheat	33.5	33.2	32.7	33.0
^5^Poultry meal	25	25	25	25
^6^Premix	1.5	1.5	1.5	1.5
^7^Rapeseed oil	4.0	4.3	4.8	4.5
Calcium phosphate	1	1	1	1

BS, black soldier fly; MW, mealworm; BSMW, 1 : 1 weight combination of black soldier fly and mealworm; Premix, vitamins, minerals, lysine, methionine, ^1^Euro-protein Ltd, Verőce, Hungary, ^2^Berg and Schmidt Pte. Ltd, Singapore, ^3^Agroloop Ltd, Rotterdam, Netherlands, ^4^ Sojaprotein, Becej, Serbia, ^5^Euro-protein Ltd, Verőce, Hungary, ^6^Cargill Ltd. Budapest, Hungary, ^7^Bunge Hungary Co. Martfű, Hungary.

**Table 2:** Proximate composition of control and experimental feeds (% as fed).

Component	Control	BS	MW	BSMW
Crude protein	44.35 ± 0.35	45.53 ± 1.09	45.11 ± 0.46	45.97 ± 0.20
Crude fat	7.95 ± 0.05	8.45 ± 0.27	8.29 ± 0.20	8.50 ± 0.06
Crude fiber	1.70 ± 0.28	3.27 ± 0.43	2.97 ± 0.11	2.95 ± 0.66
Crude ash	10.63 ± 0.21	9.38 ± 0.02	8.97 ± 0.01	8.99 ± 0.03
Gross energy (kJ g^−1^)	19.05 ± 0.04	19.94 ± 0.04	19.72 ± 0.00	19.87 ± 0.05
ADF	7.69 ± 0.05	8.62 ± 0.21	10.25 ± 0.05	10.31 ± 0.15

ADF, acid detergent fiber; BS, black soldier fly; MW, mealworm; BSMW, combination of black soldier fly and mealworm. Values are expressed in mean ± SD.

**Table 3:** Fatty acid profile (% of the total fatty acid) of the diets (as fed).

Fatty acid	Control	BS	MW	BSMW
C10 : 0	0.01	0.09	0.03	0.06
C12 : 0	0.16	4.07	0.47	2.49
C14 : 0	2.32	2.32	2.23	2.42
C14 : 1*n*5	0.04	0.05	0.04	0.05
C15 : 0	0.42	0.28	0.36	0.34
C16 : 0	17.96	14.05	16.51	16.51
C16 : 1*n*7	3.55	2.76	3.33	3.26
C16 : 2*n*4	0.09	0.60	0.09	0.09
C17 : 0	0.45	0.30	0.35	0.36
C18 : 0	4.87	3.70	4.42	4.31
C18 : 1*n*9c	36.34	31.38	37.91	36.94
C18 : 1*n*7	2.17	1.99	2.28	2.21
C18 : 2*n*6	13.12	12.31	13.80	14.28
C18 : 3*n*6	0.12	0.14	0.14	0.16
C18 : 3*n*3	2.23	2.10	2.55	2.46
C18 : 4*n*3	0.41	0.23	0.42	0.33
C20 : 0	0.32	0.27	0.35	0.32
C20 : 1*n*9	1.80	1.43	1.88	1.68
C20 : 2*n*6	0.22	0.23	0.21	0.21
C20 : 3*n*6	0.07	0.08	0.07	0.09
C21 : 0	0.03	0.03	0.02	0.03
C20 : 4*n*6	0.85	0.60	0.77	0.70
C20 : 3*n*3	0.11	0.11	0.11	0.11
C20 : 4*n*3	0.24	0.20	0.25	0.22
C20 : 5*n*3	2.68	2.00	2.64	2.36
C22 : 0	0.17	0.14	0.17	0.18
C22 : 1*n*9	0.26	0.19	0.25	0.23
C22 : 2*n*6	0.02	0.12	0.02	0.05
C22 : 5*n*3	0.71	0.55	0.68	0.64
C24 : 0	0.10	0.09	0.10	0.10
C22 : 6*n*3	7.76	5.57	7.17	6.42
C24 : 1*n*9	0.40	0.29	0.38	0.34
*n*3	14.14	10.75	13.82	12.54
*n*6	14.38	13.36	14.99	15.45
*n*6/*n*3	1.02	1.24	1.08	1.23

BS, black soldier fly; MW, mealworm; BSMW, combination of black soldier fly and mealworm; *n*-6, omega-6; *n*-3, omega-3.

**Table 4:** Slaughtering indices of African catfish and physical traits of the fillets after feeding with control, BS, MW, and BSMW diets.

Parameters	Control	BS	MW	BSMW	*p*-Value
pH 45ʹ	7.19 ± 0.20 ^*∗*^	7.15 ± 0.34	7.09 ± 0.09 ^*∗*^	7.14 ± 0.15 ^*∗*^	0.87
pH 24 hr	6.40 ± 0.13^ab^	6.61 ± 0.28^a^	6.29 ± 0.08^b^	6.12 ± 0.17^b^	0.001
Cooking loss (%)	17.19 ± 15.35	23.61 ± 11.18	19.94 ± 2.47	21.90 ± 3.10	0.69
Dripping loss (%)	2.78 ± 1.13	3.40 ± 2.35	3.05 ± 0.80	3.90 ± 0.81	0.56
Thawing loss (%)	6.06 ± 1.51	5.06 ± 3.19	6.04 ± 3.43	5.57 ± 2.05	0.80
Eviscerated slaughter value (%)	86.03 ± 9.21	91.67 ± 7.17	81.25 ± 15.81	91.19 ± 5.41	0.27
Trunk with skin (%)	60.11 ± 5.27	64.00 ± 4.28	58.48 ± 8.31	62.96 ± 3.49	0.32
Skinned trunk (%)	53.64 ± 6.21	58.47 ± 3.91	53.00 ± 7.11	57.18 ± 3.03	0.27
Filleting yield (%)	39.24 ± 6.14	44.2 ± 5.52	39.58 ± 7.28	42.12 ± 2.80	0.41
HSI (%)	0.89 ± 0.23^a^	1.33 ± 0.39^b^	0.96 ± 0.38^ab^	1.43 ± 0.44^b^	0.04
VSI (%)	1.24 ± 0.26	1.05 ± 0.08	1.21 ± 0.26	1.05 ± 0.13	0.24

BS, black soldier fly; MW, mealworm; BSMW, mixture of black soldier fly and mealworm, ^a,b^different letters between rows show a significant difference (*p* < 0.05). HSI, hepatosomatic index; VSI, viscerosomatic index. Values are expressed in mean ± SD.  ^*∗*^Significant difference between pH 45ʹ and pH 24 within one treatment.

**Table 5:** Proximate composition of African catfish fillets (% wet weight).

Parameters	Control	BS	MW	BSMW	*p*-Value
Crude protein	18.05 ± 0.85	18.82 ± 0.98	18.27 ± 0.79	18.76 ± 0.69	0.35
Crude fat	3.45 ± 2.34	3.90 ± 2.06	3.70 ± 1.34	3.53 ± 1.04	0.97
Crude ash	1.26 ± 0.09	1.27 ± 0.08	1.29 ± 0.09	1.20 ± 0.06	0.37
Moisture	76.83 ± 3.06	75.62 ± 2.70	76.31 ± 1.66	75.88 ± 1.17	0.82

BS, black soldier fly; MW, mealworm; BSMW, combination of black soldier fly and mealworm. Values are expressed in mean ± SD.

**Table 6:** Fatty acid profile (% of the total fatty acid) of the fish meat samples of different treatments.

Fatty acid	Control	BS	MW	BSMW
C12 : 0	0.52 ± 0.61^b^	1.84 ± 0.89^a^	0.25 ± 0.04^b^	0.90 ± 0.47^ab^
C14 : 0	2.15 ± 0.23	2.51 ± 0.27	2.21 ± 0.20	2.37 ± 0.26
C14 : 1*n*-5	0.03 ± 0.01	0.04 ± 0.01	0.04 ± 0.00	0.04 ± 0.00
C15 : 0	0.40 ± 0.05	0.37 ± 0.04	0.38 ± 0.06	0.37 ± 0.04
C16 : 0	25.2 ± 1.14	25.6 ± 1.37	24.2 ± 2.13	25.3 ± 1.84
C16 : 1*n*-7	3.42 ± 0.47	3.56 ± 0.65	3.36 ± 0.45	3.62 ± 0.66
C16 : 2	0.05 ± 0.01	0.05 ± 0.01	0.05 ± 0.01	0.05 ± 0.01
C16 : 3	0.03 ± 0.01	0.03 ± 0.00	0.03 ± 0.01	0.03 ± 0.01
C17 : 0	0.35 ± 0.04^a^	0.32 ± 0.03^ab^	0.29 ± 0.03^b^	0.30 ± 0.03^ab^
C18 : 0	6.19 ± 0.38	6.30 ± 0.33	7.83 ± 2.65	6.22 ± 0.67
C18 : 1*n*-7	2.64 ± 0.40	2.29 ± 0.32	2.10 ± 0.43	2.31 ± 0.34
C18 : 1*n*-9	29.9 ± 2.47	30.9 ± 1.91	31.9 ± 2.28	31.9 ± 3.09
C18 : 2*n*-6	11.3 ± 0.98	11.0 ± 1.31	12.0 ± 1.58	11.5 ± 2.30
C18 : 3*n*-3	1.26 ± 0.11	1.27 ± 0.13	1.48 ± 0.15	1.34 ± 0.30
C18 : 3*n*-6	0.47 ± 0.06	0.43 ± 0.07	0.45 ± 0.07	0.46 ± 0.13
C18 : 4*n*-3	0.25 ± 0.02^ab^	0.19 ± 0.02^b^	0.26 ± 0.05^a^	0.23 ± 0.06^ab^
C20 : 0	0.24 ± 0.04	0.23 ± 0.03	0.23 ± 0.04	0.23 ± 0.03
C20 : 1*n*-9	1.65 ± 0.22	1.64 ± 0.11	1.76 ± 0.28	1.63 ± 0.07
C20 : 2*n*-6	0.58 ± 0.13	0.48 ± 0.15	0.50 ± 0.15	0.48 ± 0.09
C20 : 3*n*-3	0.13 ± 0.03	0.12 ± 0.02	0.14 ± 0.04	0.12 ± 0.03
C20 : 3*n*-6	0.79 ± 0.08	0.74 ± 0.11	0.72 ± 0.11	0.76 ± 0.17
C20 : 4*n*-3	0.18 ± 0.02	0.17 ± 0.01	0.20 ± 0.04	0.18 ± 0.04
C20 : 4*n*-6	1.16 ± 0.27	0.96 ± 0.22	0.89 ± 0.14	0.91 ± 0.27
C20 : 5*n*-3	1.20 ± 0.18	1.07 ± 0.07	1.19 ± 0.15	1.11 ± 0.10
C21 : 0	0.02 ± 0.00	0.02 ± 0.01	0.02 ± 0.01	0.02 ± 0.01
C22 : 0	0.07 ± 0.03	0.08 ± 0.02	0.09 ± 0.03	0.09 ± 0.03
C22 : 1*n*-9	0.08 ± 0.02	0.08 ± 0.02	0.09 ± 0.02	0.08 ± 0.02
C22 : 1*n*-11	0.13 ± 0.07^a^	0.06 ± 0.04^b^	0.02 ± 0.01^b^	0.04 ± 0.03^b^
C22 : 5*n*-3	0.66 ± 0.16	0.61 ± 0.09	0.64 ± 0.10	0.60 ± 0.07
C22 : 6*n*-3	8.82 ± 2.01	6.89 ± 1.50	6.57 ± 1.16	6.66 ± 1.72
C24 : 0	0.05 ± 0.01	0.05 ± 0.01	0.05 ± 0.01	0.04 ± 0.01
C24 : 1*n*-9	0.08 ± 0.02	0.07 ± 0.03	0.07 ± 0.02	0.06 ± 0.01
Total SAT	35.2 ± 1.48	37.3 ± 1.54	35.6 ± 3.62	35.8 ± 1.79
Total UNSAT	65.7 ± 1.55	63.5 ± 1.62	65.3 ± 3.71	65.1 ± 1.87
Total MUFA	38.0 ± 2.32	38.6 ± 2.12	39.3 ± 2.54	39.7 ± 3.40
Total PUFA	27.8 ± 3.34	24.9 ± 3.25	26.0 ± 3.62	25.4 ± 4.55
Total *n*-6	14.3 ± 1.25	13.6 ± 1.76	14.6 ± 1.97	14.1 ± 2.78
Total *n*-3	12.5 ± 2.31	10.3 ± 1.57	10.5 ± 1.63	10.2 ± 1.86
*n*-6/*n*-3	1.17 ± 0.22	1.33 ± 0.12	1.40 ± 0.10	1.38 ± 0.19
Total PUFA/total SAT	0.79 ± 0.30	0.67 ± 0.25	0.73 ± 0.17	0.71 ± 0.15
Atherogenic index	0.45 ± 0.05^b^	0.57 ± 0.06^a^	0.43 ± 0.05^b^	0.49 ± 0.03^ab^
Thrombogenic index	0.52 ± 0.06	0.59 ± 0.07	0.58 ± 0.12	0.58 ± 0.09

BS, black soldier fly; MW, mealworm; BSMW, combination of black soldier fly and mealworm; *n*-6, omega-6; *n*-3, omega-3; ^a,b^ different uppercase letters in rows indicate significant difference (*p* < 0.05), values are expressed in mean ± SD.

## Data Availability

All data generated or analyzed during this study are included in this published article.
